# Peripheral Blood Mononuclear Cell Biomarkers for Major Depressive Disorder: A Transcriptomic Approach

**DOI:** 10.1155/2024/1089236

**Published:** 2024-10-03

**Authors:** Lu Sun, CaiLi Ren, HaoBo Leng, Xin Wang, DaoRan Wang, TianQi Wang, ZhiQiang Wang, GuoFu Zhang, Haitao Yu

**Affiliations:** ^1^The Affiliated Mental Health Center of Jiangnan University, Wuxi Mental Health Center, Wuxi 214151, Jiangsu, China; ^2^Department of Rehabilitation Medicine, Wuxi Central Rehabilitation Hospital, Wuxi 214151, Jiangsu, China; ^3^Wuxi Maternity and Child Health Care Hospital, Wuxi School of Medicine, Jiangnan University, Wuxi 214002, China; ^4^Department of Fundamental Medicine, Wuxi School of Medicine, Jiangnan University, Wuxi 214122, Jiangsu, China

## Abstract

**Background:** Major depressive disorder (MDD) is a complex condition characterized by persistent depressed mood, loss of interest or pleasure, loss of energy or fatigue, and, in severe case, recurrent thoughts of death. Despite its prevalence, reliable diagnostic biomarkers for MDD remain elusive. Identifying peripheral biomarkers for MDD is crucial for early diagnosis, timely intervention, and ultimately reducing the risk of suicide. Metabolic changes in peripheral blood mononuclear cells (PBMCs) have been observed in animal models of depression, suggesting that PBMC could serve as a valuable matrix for exploring potential peripheral biomarkers in MDD.

**Methods:** We performed a transcriptomic analysis of PBMCs from patients with MDD and age- and sex-matched healthy controls (*n* = 20 per group).

**Results:** Our analysis identified 270 differentially expressed genes in PBMCs from MDD patients compared to controls, which correlated with the Hamilton Depression Rating Scale scores. These genes are involved in several KEGG pathways, including the herpes simplex virus 1 infection pathway, NOD-like receptor signaling pathway, antigen processing and presentation, and glycerophospholipid metabolism—all of which are linked to various aspects of the immune response. Further machine learning analysis and quantitative real-time PCR (qPCR) validation identified three key genes—TRPV2, ZNF713, and CTSL—that effectively distinguish MDD patients from healthy controls.

**Conclusions:** The immune dysregulation observed in PBMCs is closely related to the pathogenesis of MDD. The candidate biomarkers TRPV2, ZNF713, and CTSL, identified and validated through machine learning and qPCR, hold promise for the objective diagnosis of MDD.

**Trial Registration:** Clinical Trial Registry identifier: ChiCTR2300076589

## 1. Introduction

Major depressive disorder (MDD) is a complex condition characterized by persistent symptoms including depressed mood, loss of interest or pleasure, recurrent suicidal thoughts, and cognitive impairments [1]. According to statistics published by the World Health Organization in 2019, MDD affects over 280 million people worldwide [2–4] and remains the leading cause of disability, economic burden, and mortality [5, 6]. Early intervention can significantly improve treatment outcomes, making timely and accurate diagnosis essential [7]. Currently, clinical interviews are the primary method for diagnosing MDD, as there are no established biochemical markers for routine diagnosis and treatment [8]. This highlights the urgent need for noninvasive reliable biomarkers in MDD.

Recent studies have explored the relationship between immune system disorders and MDD [9, 10]. For instance, research has identified a stress-induced increase in a specific protease, matrix metalloproteinase 8 (MMP8), in peripheral circulating myeloid cells. This increase is associated with changes in the extracellular space of the nucleus ambiguus, which can impact neurophysiology and social behavior [11]. Peripheral blood mononuclear cells (PBMCs) have been showed to correlate with depression symptoms [12]. Clinical trials suggest that chronic stress stimulates the bone marrow to produce and transport PBMCs to the central nervous system, where they can worsen the release of pro-inflammatory factors, ultimately contributing to depressive-like behavior [13, 14]. Studies using the chronic unpredictable stress model have demonstrated an increase in PBMCs within the bone marrow, leading to elevated levels of PBMCs in the bloodstream [15]. Additionally, repeated social deprivation in animal models promotes the migration of PBMCs from the bone marrow into circulation, while antidepressant treatments can inhibit this release [16–18]. Similar findings have been observed in patients with MDD, who exhibit higher levels of PBMCs and altered cerebral white matter recruitment [19, 20]. Furthermore, recent research suggests that MDD is associated with morphological changes in immune cell, including increased deformability of PBMCs [21]. Together, these findings underscore the importance of PBMCs as potential peripheral biomarkers for depression.

Transcriptomics, the study of genome-wide mRNA expression, play a crucial role in understanding gene expression and identifying biomarkers for various diseases [22–24]. By comparing gene expression profiles between normal and disease states, researchers can identify differentially expressed genes (DEGs) that may serve as biomarkers [25]. This approach has diverse applications in biomarker screening. In this study, we employed RNA sequencing (RNA-Seq) to conduct a comprehensive transcriptomic analysis of PBMCs from individuals diagnosed with MDD, comparing them to age- and sex-matched healthy controls. In this study, we utilized RNA-Seq to obtain the expression profile of PBMCs from patients diagnosed with MDD. We further identified DEGs that are correlated with the Hamilton depression rating scale (HAMD) and conducted pathway enrichment analysis based on these genes, revealing that MDD is associated with immune response-related pathways such as the herpes simplex virus 1 infection pathway, NOD-like receptor signaling pathway, antigen processing and presentation, and glycerophospholipid metabolism. Employing a stringent feature selection strategy, we “locked in” on three pivotal genes and validated them through quantitative real-time PCR (qPCR). We identified a set of biomarkers, namely TRPV2, ZNF713, and CTSL, which are anticipated to objectively predict MDD and may offer new insights into the pathogenesis of MDD.

## 2. Materials and Methods

### 2.1. Participant Inclusion and Exclusion Criteria

The study included 20 patients diagnosed with MDD at Wuxi Mental Health Center and 20 healthy controls from the community and university, matched by age and gender. The diagnostic criteria adhered to the Fourth Edition of the Diagnostic and Statistical Manual of Mental Disorders (DSM-IV). Participants included both men and women aged 18–60 with a HAMD score of 17 or higher. Exclusion criteria included individuals with recent or chronic inflammatory conditions, autoimmune disorders, acute physical illnesses, or substance abuse. Additionally, healthy controls were excluded if they had a personal or familial history of psychiatric disorders. Clinical assessments were performed by trained professionals, as detailed in Table 1.

The research was approved by the Ethics Committee of Wuxi Mental Health Center (protocol number WXMHCIRB2023LLky073), with written consent was obtained from all participants.

### 2.2. Isolation of PBMCs

To isolate PBMCs, 2 ml of recently drawn whole blood were mixed with 2 ml of phosphate-buffered saline (PBS). This mixture was gently combined and then layered with 3 ml of Ficoll–Paque PLUS in a centrifuge tube. The tube was centrifuged at 3000 g for 30 min at a temperature of 18°C−20°C. After centrifugation, the mononuclear cell layer was carefully removed and washed. The cells were allowed to settle for 10 min at a speed of 60–100 g at the same temperature. The supernatant was discarded, and TRIzol was added to preserve the cells, which were then stored at −80°C until further analysis.

### 2.3. RNA Extraction

Total RNA was extracted from the monocytes using TRIzol reagent (Magen). The RNA integrity number (RIN) values were assessed by calculating the A260/A280 absorbance ratio of the samples using a Nanodrop ND-2000 (Thermo Scientific, USA) and analyzing them on an Agilent Bioanalyzer 4150 (Agilent Technologies, CA, USA). Only RNA samples that met quality control criteria were selected for library preparation.

### 2.4. Library Preparation and Sequencing

The paired-end (PE) library was prepared according to the guidelines for the ABclonal mRNA-seq Lib Prep Kit (ABclonal, China). First, 1 μg of total RNA was used to extract mRNA using oligo (dT) magnetic beads. The mRNA was then fragmented in the ABclonal first strand synthesis reaction buffer. Next, the first cDNA strand was synthesized from the mRNA fragments using random primers and reverse transcriptase (RNase H). Subsequently, the second cDNA strand was synthesized with DNA polymerase I, RNase H, buffer, and dNTPs. The double-stranded cDNA was amplified by ligating the junction sequence. The obtained PCR products were purified, and the Agilent Bioanalyzer 4150 was used to evaluate the quality of the library. Finally, sequencing was performed on the Illumina Novaseq 6000/MGISEQ-T7 platform.

### 2.5. Data Preprocessing

For high-throughput sequencing analysis, the raw image data files were initially analyzed using CASAVA software (version 1.8) for base recognition, transforming into raw sequencing sequences in FASTQ format. To ensure the accuracy of the subsequent analysis, these sequences underwent rigorous quality control, which included the removal of splice sequences and filtering out low-quality reads. This process resulted in high-quality clean reads. The clean reads were then aligned with the reference genome using HISAT2 (version 2.2.1) software, allowing for precise positioning on the genome. Gene expression levels were assessed by measuring transcript abundance. We employed the featureCounts (version 2.0.3) software to calculate the fragments per kilobase of transcript per million mapped reads(FPKM) value for each gene, providing a standardized expression metric that facilitated effective comparisons across samples and genes.

### 2.6. Bioinformatics Analysis

Differential expression analysis of genes between groups was conducted using the DESeq2 package (version 1.42.0), with default screening thresholds for DEGs set at |log2FC| > 0.5 and *Padj* <0.05. Cluster analysis and heatmap plotting were performed using R Studio (version 4.3.2) and heatmap plot packages (version 1.0.12). Volcano maps were drawn using GraphPad Prism (version 9.5). Metascape (http://metascape.org) was used to perform clustering, pathway, and biofunctional enrichment analyses [26]. The interactions between proteins corresponding to genes were obtained using the STRING database (https://www.string-db.org/). Cytoscape and MCODE (composite molecular plug-in) were used to visualize protein interaction network modules.

### 2.7. Machine Learning

We employed three machine learning algorithms—LASSO, random forest (RF), and support vector machine recursive feature elimination (SVM-RFE)—to comprehensively screen for candidate genes associated with MDD. All analyses were conducted using R Studio (version 4.3.2). LASSO regression, which incorporates L1 regularization, effectively performed variable selection by reducing the coefficients of nonessential genes to zero while maintaining the predictive accuracy of the model. The glmnet package (version 4.1.8) was utilized to implement the LASSO algorithm, and 10-fold cross-validation was employed to identify significant genes. SVM-RFE combines the classification power of SVM with recursive feature elimination to identify key genes. We used the e1071 package (version 1.7.14) to implement this method, determining the optimal number of genes based on their accuracy. RF is an ensemble learning method that enhances prediction accuracy and robustness by constructing numerous decision trees. Additionally, RF assesses feature importance, aiding in identifying the most influential features for prediction, which assists in feature selection and model interpretation. The RF algorithm was executed using the randomForest package (version 4.7.1.1). Finally, candidate genes associated with MDD were identified by cross-referencing results from the three machine learning methods. We employed the pROC software package (version 1.18.5) to evaluate the discriminatory ability of both single and combined gene diagnostic models, quantifying diagnostic value by calculating the area under the curve (AUC). An AUC exceeding 0.7 was considered indicative of ideal diagnostic performance [27].

### 2.8. qPCR

The research involved a total of 50 participants diagnosed with MDD and an equal number of healthy controls. RNA extraction from PBMCs was performed using TRIzol reagent. To validate the DEGs identified in the transcriptome data, qPCR was conducted. GAPDH was utilized as the internal reference gene, and the relative mRNA expression of the identified genes was determined using the 2^−*ΔΔ*Ct^ method. Validation focused on interleukin-8 (CXCL8), transient receptor potential vanilloid 2 (TRPV2), tissue protease L (CTSL), proteasome activator complex subunit 2 (PSME2), and zinc finger protein 713 (ZNF713) in *Homo sapiens*. Primer design was carried out using Primer 5 software, and synthesis was conducted by Shanghai Jiman Biologicals Co. The primer sequences can be found in Table 2.

### 2.9. Statistical Analysis

Statistical analyses were conducted using SPSS version 26.0 (Statistical Program for the Social Sciences, Inc.). The Shapiro–Wilk test was performed to assess the normality of the data. The Mann–Whitney *U* test was applied for non-normally distributed data. In contrast, for data that were normally distributed, we utilized the two-independent samples *t* test to evaluate the differences in means between the two groups. For the correlation analysis with HAMD scores, the Spearman correlation analysis was employed. The threshold for statistical significance was set at *p* < 0.05.

## 3. Results

### 3.1. Differential Genes and Pathways in MDD and Healthy Control PBMCs

We conducted a study involving 20 patients diagnosed with MDD, with HAMD scores ranging from 17 to 33. We also included 20 healthy controls, age- and sex-matched, with HAMD scores ranging from 0 to 6. The primary objectives of this study were to collect transcriptome data from PBMCs and to perform a bioinformatics analysis. The primary objectives of this study were to collect transcriptome data from PBMCs and perform a bioinformatics analysis. Specifically, the study aimed to: (a) analyze the differences in PBMC transcriptional profiles between patients with MDD and healthy controls; (b) elucidate the biological mechanisms associated with PBMCs in MDD; (c) identify gene correlated with HAMD scores; and (d) discover peripheral blood biomarkers of MDD.

**Figure 1:**
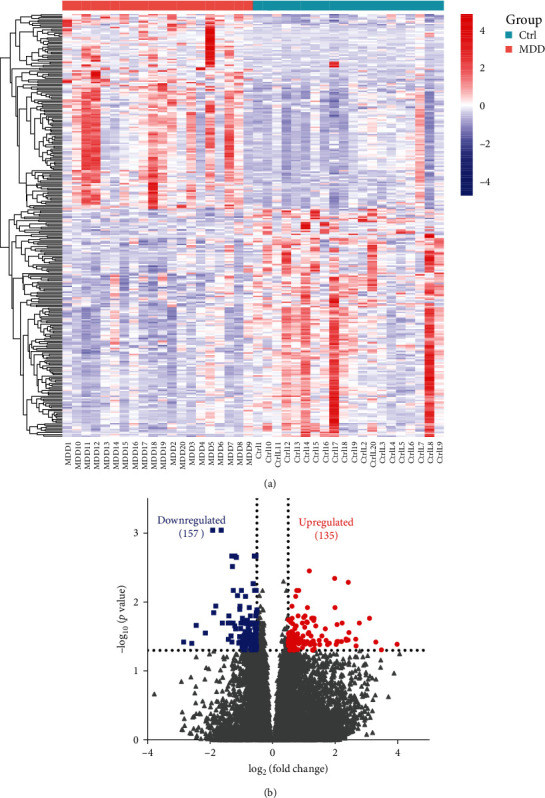
Differential genes in PBMCs of MDD patients and healthy control. (a) Differential gene expression changes and their relative abundance in patients with MDD compared to healthy controls. (b) A total of 292 species were identified in PBMCs from patients with MDD versus healthy controls (*padj* <0.05, |log2 (FoldChange)| > 0.5; gene increase: red; gene decrease: blue).

A total of 38,960 genes were captured using Illumina transcriptome analysis, of which 292 genes showed significant differences between MDD patients and healthy controls (padj <0.05, |log2 (fold change) | > 0.5;Figure 1a). Of these, 135 genes were upregulated, while 157 genes were downregulated (Figure 1b).

**Figure 2:**
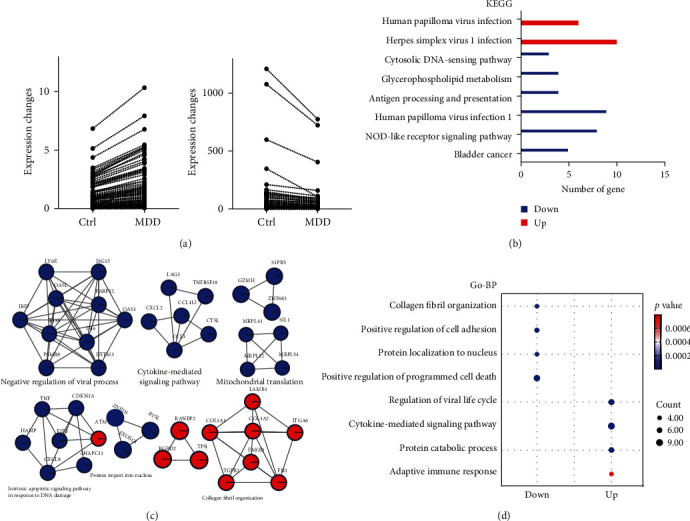
Differential genes and biological pathways were found in MDD patients compared to normal controls. (a) Changes in the expression of differentially up and downregulated genes, with each line representing a specific gene. (b) Pathway enrichment analysis of up and downregulated genes using Metascape online analysis, where significantly enriched pathways are defined as having overlapping proteins ≥3, *p* < 0.01). (c) Protein–protein (PPI) modules are detected in clusters, where blue and red colors represent down- and upregulated genes, respectively. (d) Biological function enrichment analysis of differentially expressed genes, where up and down represent up and downregulated genes, respectively.

To investigate changes in the PBMC transcriptome in MDD, genes were categorized into upregulated and downregulated groups based on a differential analysis comparing MDD patients with healthy controls (Figure 2a). The upregulated genes (*n* = 135) included ATM, COL1A1, FN1, ITGA6, and LAMB1, which were found to be enriched in pathways related to human papillomavirus infection and herpes simplex virus 1 infection (Figure 2b). On the other hand, the downregulated genes (*n* = 157), such as ISG15, IRF7, OASL, OAS1, TNF, CDKN1A, CXCL8, E2F1, CXCL2, CTSL, and CCL4L2, were enriched in pathways related to bladder cancer, Nod-like receptor signaling, human papillomavirus infection, antigen processing and presentation, glycerophospholipid metabolism, and cytoplasmic DNA- sensing (Figure 2b). Additionally, a protein–protein interaction (PPI) network analysis using MCODE revealed 8 protein interaction modules supporting the identified pathways for the DEGs (Figure 2c). Further analysis of the biological processes associated with these genes uncovered detailed mechanisms such as regulation of viral life cycle, adaptive immune response (downregulation), and positive regulation of programmed cell death, positive regulation of cell adhesion, collagen fibril organization, and protein localization in the nucleus (upregulation;Figure 2d). These transcriptome-wide data reveal that differential genes and associated pathways in MDD patients and healthy controls are strongly focused on immune function.

### 3.2. Spearman Analysis of Differential PBMC Genes or Pathways Associated With HAMD Scores

**Figure 3:**
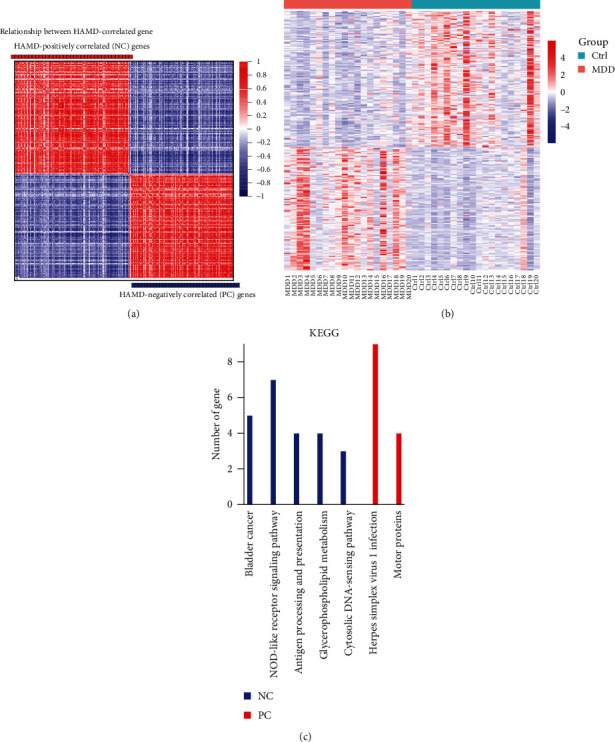
PBMC transcriptome differential genes correlated with HAMD scores. (a) Spearman analysis of HAMD score negatively correlated (NC, blue) and positively correlated (PC, red) genes (*p* < 0.05), and ranked according to their coefficients. (b) Heat map depicting the relative abundance of all HAMD-related genes in each sample, with increasing genes expression shown in red and decreasing gene expression shown in blue. (c) KEGG pathways enriched for all HAMD-associated genes, with the significance of enriched pathways defined as overlapping genes ≥3 and *p* < 0.01.

The HAMD is the most prevalent clinical scale for rating depressive states. To explore peripheral biomarkers that can objectively predict MDD, we performed a correlation analysis of HAMD scores with the entire transcriptomics data of normal healthy controls and MDD. A total of 270 genes were identified as highly correlated (*p* < 0.05) with HAMD scores, including 142 negatively correlated (NC) genes and 128 positively correlated (PC) genes (Figures 3a,b). The NC genes were enriched in multiple pathways including bladder cancer, NOD-like receptor signaling pathway, antigen processing and presentation, glycerophospholipid metabolism, and cytoplasmic DNA-sensing pathway (Figure 3c), while the PC genes were significantly enriched in pathways related to herpes simplex virus type 1 infection and motor proteins (Figure 3c).

**Figure 4:**
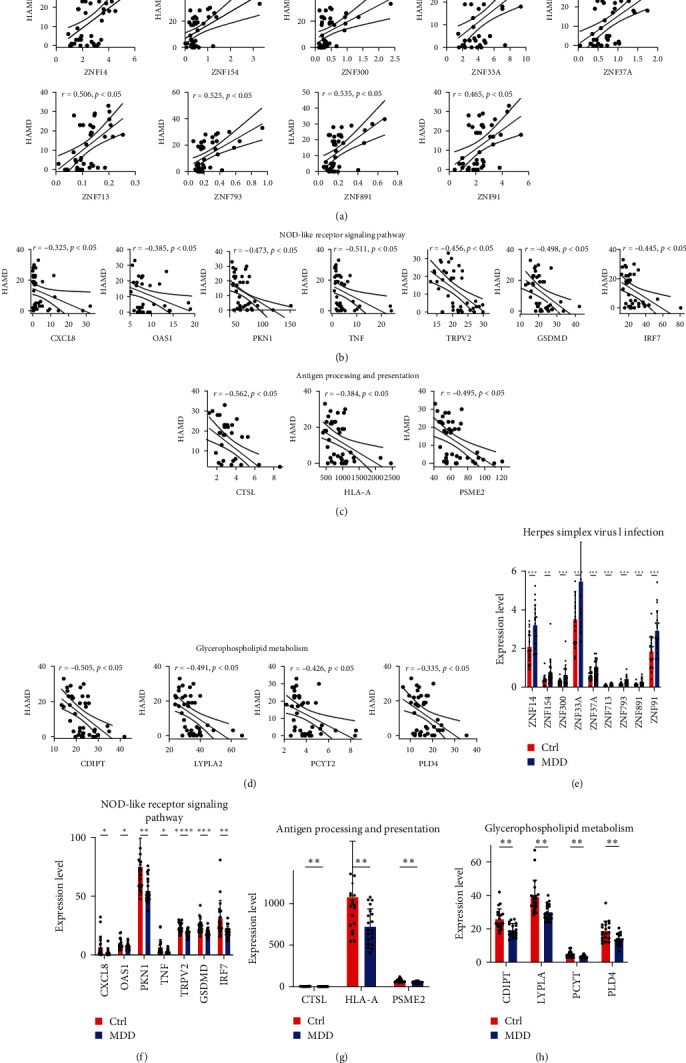
Integration and quantitative analysis of HAMD-related pathways and DE genes. (a) Genes of the KEGG signaling pathway were positively correlated with the HAMD scale with herpes simplex virus type I infection. (b–d) Genes enriched in the KEGG signaling pathway were negatively correlated with the HAMD scale with the Nod-like receptor signaling pathway, glycerophospholipid metabolism, and antigen processing and presentation. Pearson correlation coefficients (*r*) and the corresponding *p* < 0.05, are shown at the top of each graph. the *x*-axis indicates the HAMD scores, and the *y*-axis indicates the relative expression abundance of each gene. (e–h) plots indicate the relative expression levels of each gene in different samples. Data are expressed as SEM ± mean. ⁣^*∗*^*p* < 0.05, ⁣^*∗∗*^*p* < 0.01, ⁣^*∗∗∗*^*p* < 0.001, and *⁣*^*∗∗∗∗*^*p* < 0.0001.

**Figure 5:**
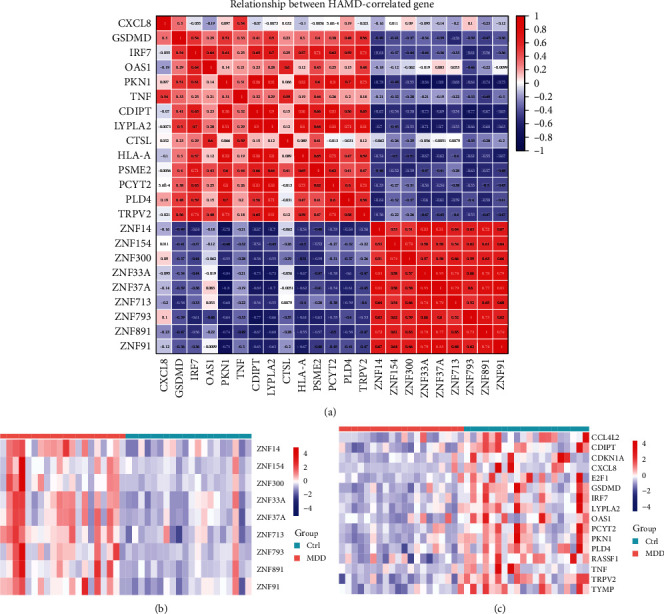
Ranking of correlations between candidate gene levels and HAMD scores. (a) Negative (blue) and positive (red) correlation candidate biomarkers (*p* < 0.05) are ranked according to their Pearson correlation coefficients. The color depth scale indicates the degree of correlation. (b–c) The relative abundance of the correlated genes, where (b) shows the relative abundance of positively correlated genes, and (c) shows the relative abundance of negatively correlated genes.

By further integrating the differential genes from the whole transcriptome with HAMD-related genes, we identified four KEGG pathways, including one PC pathway and three NC pathways, which are strongly associated with HAMD-related pathomechanisms (Figures 4a–d). Twenty-three genes involved in herpes simplex virus type I infection, Nod-like receptor signaling, antigen processing and presentation, and glycerophospholipid metabolism were not only genetically different but also associated with HAMD scores (Figures 4a–f). Specifically, the genes involved in transcriptional regulation included ZNF33A, ZNF37A, ZNF91, ZNF154, ZNF713, ZNF793, and ZNF891, along with ZNF300, which exhibits transcriptional deterrent protein activity (Figures 4a,e). Additionally, neutrophil chemokine CXCL8, interferon-regulated transcription factor IRF7, interferon-induced dsRNA-activated antiviral enzyme OAS1, protein kinase N1 (PKN1), and TNF, which are essential for host defense and inflammation, TRPV2 and GSDMD play important roles in protecting the host from the infection of pathogens and signaling of danger (Figures 4b,f). The glycerophospholipid metabolism pathway included genes such as PCYT2, CDIPT, LYPLA2, and PLD4 (Figures 4c,g). Moreover, CTSL, involved in general protein degradation within lysosomes, HLA-A, which presents intracellular antigens to the cell surface, and PSME2, which is essential for antigen processing, were also identified (Figures 4d,h). By further ranking the correlation coefficients of the above 23 PBMC candidate genes with the HAMD score, complex associations were identified (Figures 4a–d), and the increase or decrease in these candidates was well uniform in each group (Figures 4e–h). All 23 candidate genes showed a correlation with HAMD (|*r*| = 0.325–0.562; Figures 4a–d). Among them, the increase in ZNF37A, a gene involved in transcriptional regulation, had the strongest correlation with the HAMD score (|*r* | = 0.562, *p*  < 0.05; Figure 4a). It is noteworthy that a significant correlation was observed between certain genes within the dataset, including those involved in transcriptional regulation (ZNF33A, ZNF37A, ZNF91, ZNF154, ZNF713, ZNF793, ZNF891) and ZNF300, which has transcriptional repressor protein activity (|*r*| = 0.51–0.95); glycerophospholipid metabolism PCYT2, CDIPT, PLD4 (|*r*| = 0.6–0.83); neutrophil chemotactic factor CXCL8 and TNF critical for host defense and inflammation (|*r*| = 0.54); IRF7, GSDMD (|*r*| = 0.54) regulated by interferon-regulated transcription factors play an important role in protecting the host from infection and danger signals; HLA-A is critical for intracellular antigen presentation to the cell surface and antigen processing. PSME2 is important (|*r*| = 0.65); CTSL is involved in the overall degradation of proteins in lysosomes, and PSME2 is critical for antigen processing (|*r*| = 0.41). PKN1, glycerophospholipid metabolism-related gene LYPLA2 and ZNF33A involved in transcriptional regulation, and HLA-A that presents intracellular antigens to the cell surface (|*r*| = 0.84–0.91) (Figure 5a). It is suggested that the genes on these four pathways may play a coordinating or antagonistic role in the pathogenesis of MDD. Together, these data suggest that these immune-related genes regulate complex networks associated with MDD.

### 3.3. Screening and Validation of PBMC Biomarkers

**Figure 6:**
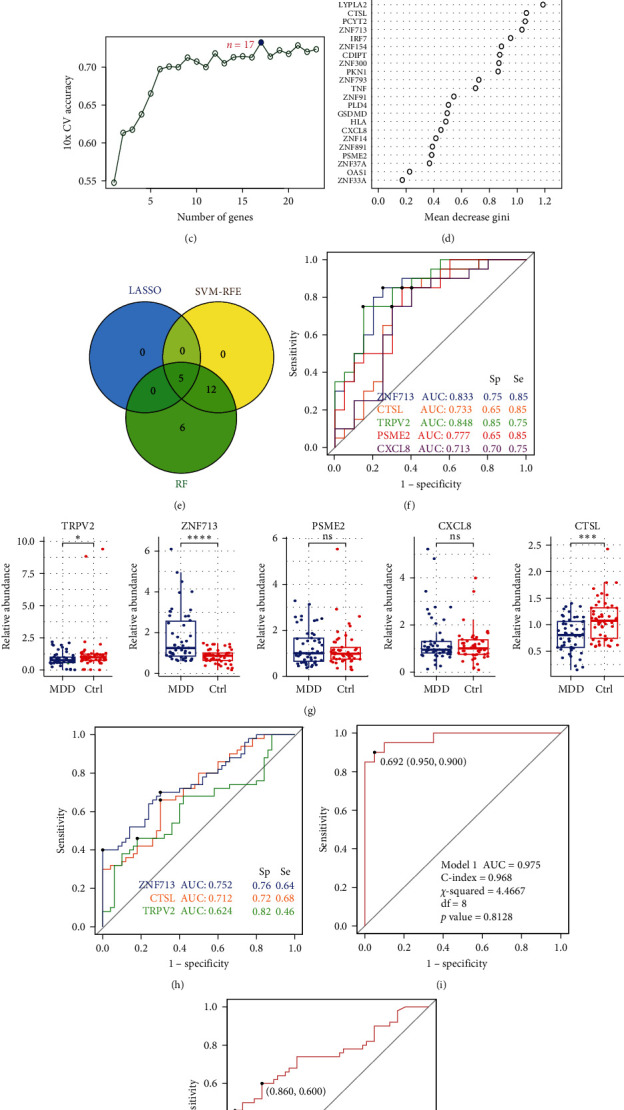
Screening and validation of biomarkers. (a–e) Five circulating differential genes were identified as diagnostic biomarkers by LASSO regression, SVM-RFE, and RF methods. (f) ROC curves were evaluated to assess the diagnostic efficacy of these 5 genes: CTSL (AUC = 0.733), PSME2 (AUC = 0.777), ZNF713 (AUC = 0.833), CXCL8 (AUC = 0.713), TRPV2 (AUC = 0.848). (g) qPCR was employed to validate the differential expression of five genes between the two groups. (h) ROC profiles were evaluated to assess the diagnostic efficacy of three selected genes: CTSL (AUC = 0.712), TRPV2 (AUC = 0.624), and ZNF713 (AUC = 0.752). (i–j) Diagnostic performance of a three-gene signature was analyzed in both discovery and validation cohorts using ROC analysis. Specificity = [true negative/(true negative + false positive)]. Sensitivity = [true positive/(true positive + false negative)].

Five differential genes were selected from the initial 23 differential genes using LASSO regression (Figures 6a,b), while an additional 17 differential genes were identified using SVM-RFE, achieving maximal accuracy of 0.764 (Figure 6c). The importance of these genes was ranked using RF analysis (Figure 6d). Ultimately, five biomarkers were identified: CTSL, PSME2, ZNF713, CXCL8, and TRPV2 (Figure 6e). The ROC curve analysis indicated the following performance for each biomarker: CTSL (AUC = 0.733, specificity: 0.65, sensitivity: 0.85), PSME2 (AUC = 0.777, specificity: 0.65, sensitivity: 0.85), ZNF713 (AUC = 0.833, specificity: 0.75, sensitivity: 0.85), CXCL8 (AUC = 0.713, specificity: 0.70, sensitivity: 0.75), and TRPV2 (AUC = 0.848, specificity: 0.85, sensitivity: 0.75) (Figure 6f). In the validation cohort, analyzed by qPCR, we observed a decreasing trend in the levels of CTSL and TRPV2 in MDD patients and an increasing trend in ZNF713, which was consistent with the transcriptomic results (Figure 6g). ROC curve analysis revealed that ZNF713 had an AUC of 0.752, a specificity of 0.76, and a sensitivity of 0.64; CTSL had an AUC of 0.712, a specificity of 0.72, and a sensitivity of 0.68; TRPV2 had an AUC of 0.624, a specificity of 0.82, and a sensitivity of 0.46 (Figure 6h).

In the discovery cohort, the diagnostic model for these three genes demonstrated high diagnostic value, with an area under the ROC curve (AUC) of 0.975 and a C-index of 0.968. The model's goodness-of-fit was assessed using the Hosmer–Lemeshow test, which yielded a *χ^2^* value of 4.467 with degrees of freedom = 8 and a *p* value of 0.8128, indicating no significant difference between the observed and expected outcomes (Figure 6i).

Upon validation in the independent cohort, the model maintained a respectable performance, with an AUC of 0.756 and a C-index of 0.739. The Hosmer–Lemeshow test for model fit returned a *χ^2^* value of 4.7793, with 8 degrees of freedom, and a *p* value of 0.7809, suggesting that the model continues to fit the data well (Figure 6j).

In summary, our study provides valuable insights into the molecular characterization of depression and lays the groundwork for future research aimed at developing novel diagnostic approaches. The discovery and validation of TRPV2, ZNF713, and CTSL as potential biomarkers offer a promising avenue for enhancing the precision of depression diagnosis and advancing our understanding of this complex disorder.

## 4. Discussion

MDD is a leading cause of disability and the primary contributor to suicide worldwide, highlighting its public health concern [28]. The underlying mechanisms of depression remain unclear, and there is a lack of definitive diagnostic tools. As a result, the identification of objective peripheral biomarkers is crucial for improving the diagnosis and treatment of depression.

Our study has revealed that the gene functions associated with MDD are predominantly concentrated on pathways related to immunity. This includes the herpes simplex virus 1 infection pathway, the NOD-like receptor signaling pathway, antigen processing and presentation, and glycerophospholipid metabolism. These findings are consistent with previous studies, suggesting that patients with MDD may have an imbalance in immune responses. Furthermore, our research has identified a new set of noninvasive potential diagnostic biomarkers for MDD, namely TRPV2, ZNF713, and CTSL. The identification of these biomarkers was completed through blood sampling, a process that is both low-risk and cost-effective. We identified these biomarkers in our initial study cohort and validated them in an independent cohort, finding that these combinations of monocyte biomarkers have significant diagnostic potential. These results not only enrich our understanding of the pathophysiological mechanisms of MDD but also provide new directions for future diagnosis and treatment.

Among the identified biomarkers, CTSL emerges as a potential diagnostic biomarker for MDD. Cathepsin L is a lysosomal cysteine protease involved in various biological processes, including protein degradation and antigen presentation [29], which are then presented to the immune system to activate specific immune responses [30, 31]. Given the potential role of inflammatory responses, CTSL might influence the onset of MDD by modulating immune processes. Our findings indicate reduced CTSL expression in patients with MDD, which could impair immune function and contribute to depression. Additionally, CTSL plays a crucial role in tumor biology, particularly in the processes of invasion and metastasis by degrading the extracellular matrix and basement membrane, thus promoting tumor cell invasion and dissemination [32, 33]. Our study found that DEGs are significantly enriched in the bladder cancer signaling pathway. Depression is common among bladder cancer patients and may exacerbate tumor growth by affecting immune-related cells and molecules [34]. Recent studies have revealed a close association between CTSL and bladder cancer [35, 36]. The reduced CTSL level observed in our study. It may affect tumor-associated inflammation and progression, suggesting a complex interplay between CTSL, depression, and cancer [37].

ZNF713 is a protein with C2H2 zinc finger domains that plays a role in regulating gene transcription and has been linked to neurodevelopmental disorders. Previous research has identified a CGG repeat expansion mutation in the 5' intron region of the ZNF713 gene in some patients with neurodevelopmental disorders, potentially altering gene expression and function, and being associated with Autism Spectrum Disorder (ASD) [38]. A multitude of zinc finger protein members are key regulators of immune responses. For example, ZNF683 is involved in the regulation of effector T cells and NK cells, thereby influencing immune memory and response [39]; Zfp281 plays a key role in the immune response by directly suppressing the expression of CTLA-4, which negatively regulates T cell activation and cytokine production [40]. Therefore, we speculate that ZNF713 may play a role in the immune response through a similar mechanism. Although the potential roles of ZNF713 in neurodevelopmental disorders and immune responses are of interest, no studies have reported the relationship between ZNF713 and depression. Future research should further explore the function of ZNF713 and its specific roles in the nervous and immune systems.

TRPV2, a calcium-permeable nonselective cation channel, plays a key role in synaptic plasticity, essential for mood regulation [41, 42]. Chronic stress has been shown to be associated with reduced TRP2 expression in the hippocampus, a brain region critical for mood regulation [43]. This reduction might impair synaptic function, a characteristic commonly observed in individuals with depression [44]. Activating TRPV2 may enhance synaptic plasticity in hippocampal neurons and potentially mitigate depressive symptoms [45]. Brain-derived neurotrophic factor (BDNF) is a neurotrophic factor closely related to the plasticity of the nervous system, the survival of neurons, and synaptic function, playing a significant role in the pathogenesis and treatment of depression [46, 47]. Moreover, TRPV2 influences the ERK1/2-CREB-BDNF signaling pathway, which is involved in neuroplasticity and depression [48].

## 5. Limitations

Our study might provide valuable insight into potential biomarkers for depression, but it has limitations. The relatively small sample size may limit the statistical power, potentially increasing the risk of false-negative or false-positive results. However, we validated these identifying biomarkers in an independent cohort. Future studies with large sample sizes are needed to validate these findings and improve the generalizability of our results. Additionally, while promising, the clinical application of these biomarkers requires further investigation. We will pursue follow-up studies to explore the potential of these genes in clinical diagnosis and treatment.

Despite these limitations, our research contributes to understanding the biological mechanisms of depression and lays the groundwork for developing new diagnostic tools and therapeutic strategies.

## 6. Conclusions

Through comprehensive bioinformatics analysis, it has been observed that the biological pathways in peripheral PBMCs of patients with depression are predominantly focused on immune pathways, which may be strongly associated with the pathogenesis of MDD. Utilizing machine learning algorithms for the selection of potential biomarkers, such as LASSO, SVM-RFE, and RF, followed by validation through qPCR in a validation cohort, we have identified TRPV2, ZNF713, and CTSL as potential biomarkers for MDD. These genes not only offer promising directions for future research but also may serve as potential therapeutic targets for the management of depression.

## Figures and Tables

**Table 1:** Comparison of demographics between MDD and Ctrl groups.

Characteristic	MDD (*n* = 20)	Ctrl (*n* = 20)	t/*χ*^2^/z	*p*-Value
Gender (male/female)	10/10	10/10	0.000	1
Age (years) (mean ± SD)	34.0 ± 2.6	34.5 ± 2.6	−0.134	0.894
Education (years) (median (lower quartile to upper quartile))	12.5 (9–16)	15 (15–17)	−1.927	0.060
HAMD-17 Score (median (lower quartile to upper quartile))	22.5 (18.75–27.25)	1 (0−3)	4.977	<0.001

Abbreviations: Ctrl, healthy control; HAMD, Hamilton Depression Rating Scale; MDD, major depressive disorder.

**Table 2:** PCR primers employed in the present study.

Gene	Forward primer (5′ → 3′)	Reverse primer (5′ → 3′)
CXCL8	ACTGAGAGTGATTGAGAGTGGAC	AACCCTCTGCACCCAGTTTTC
TRPV2	TCAGGTTGGAGACATTAGATGGA	TCAGGTTGGAGACATTAGATGGA
CTSL	CACCGGCTTTGTGGACATC	ATGACCTGCATCAATAGCAACA
PSME2	TTTGGGGTAGCAATCCAGGAG	CCAAGGCCCGGTAATCCAT
ZNF713	TTCACCAGAGAGGAGTGGGA	TACAAAGCTGATACCCCAGTGC
GAPDH	GGTCATCATCTCTGCCCCCTCT	ACAGTCTTCTGGGTGGCAGTGATG

## Data Availability

The original data for this study has been uploaded to a database. Once our data is published, you can access our SRA records through the following link: https://www.ncbi.nlm.nih.gov/bioproject/PRJNA1137251/.
